# Relationship of epidural patient-controlled analgesia with postoperative bleeding after unilateral total knee arthroplasty: a propensity score-matching analysis

**DOI:** 10.1038/s41598-021-90946-5

**Published:** 2021-05-28

**Authors:** Kyung-Don Hahm, Seok-Joon Jin, Seong-Sik Cho, Jihoon Park, Han Park, Doo-Hwan Kim, Seong-Soo Choi

**Affiliations:** 1 Department of Anesthesiology and Pain Medicine, Asan Medical Center, University of Ulsan College of Medicine, 88 Olympic-ro 43-gil, Songpa-gu, Seoul 05505 Korea; 2 Department of Pain, Shinshinplus Clinic, Anyang-si, 14401 Korea; 3 Department of Occupational and Environmental Medicine, College of Medicine, Dong-A University, Busan, 49201 Korea

**Keywords:** Outcomes research, Osteoarthritis

## Abstract

Although epidural patient-controlled analgesia (PCA) to control postoperative pain after total knee arthroplasty (TKA), the relationship of epidural PCA with postoperative bleeding remains controversial. Therefore, we aimed to evaluate the effect of epidural and intravenous PCA on postoperative bleeding in patients undergoing unilateral TKA. Total of 2467 patients who underwent TKA were divided to intravenous PCA (n = 2339) or epidural PCA (n = 128) group. After 1:1 propensity score-matching, 212 patients were analyzed to assess the associations between the perioperative blood loss and epidural PCA between the groups. Mean postoperative blood loss was significantly greater in epidural PCA than in intravenous PCA (900.9 ± 369.1 mL vs. 737.8 ± 410.1 mL; *P* = 0.007). The incidence of red blood cell (RBC) administration (> 3 units) was significantly higher in epidural PCA than in intravenous PCA (30.2% vs. 16.0%; OR 2.5; 95% CI 1.201–5.205; *P* = 0.014). Epidural PCA may be strongly related to postoperative bleeding and the incidence of RBC transfusion of more than 3 units after unilateral TKA, as compared to intravenous PCA. Therefore, the use of epidural PCA may be carefully considered for postoperative pain management in TKA.

## Introduction

Total knee arthroplasty (TKA) is usually associated with serve postoperative pain due to the occurrence of the extensive surgical trauma of the muscle and bone tissue, as well as tourniquet compression and decompression of the operated leg. The inadequate control of postoperative pain may result in chronic postoperative pain and poor outcomes^1–3^. Epidural pain management yields superior pain relief as compared with parenteral regimens during the postoperative period^4^. In patients who have undergone TKA, epidural patient-controlled analgesia (PCA) or continuous nerve blocks leads to the quicker application of intense physical therapy—the most fundamental factor of good postoperative knee rehabilitation—as compared with intravenous PCA^5, 6^. The prevention of blood loss during and after knee surgery is important, as the incidences of respiratory tract infection and wound infection are reported to be significantly greater in patients receiving allogeneic blood transfusions, compared with those receiving no blood transfusion^7^. Moreover, transfused patients are more likely to have greater in-hospital mortality, hospital stay, and total costs per admission^8^.

Previous studies have described the relationship between regional anesthesia and decreased blood loss during orthopedic surgery^9–12^. However, the effects of epidural PCA on postoperative bleeding remain controversial. Furthermore, most studies have limitations such as unbalanced demographics and intraoperative variables between groups, or an inadequate power to evaluate the differences between groups. In particular, significant predictors for transfusion, such as preoperative hemoglobin, age, female gender, body mass index, creatinine, intraoperative blood loss, and intraoperative fluids, should be controlled before comparison^13^. In the present study, we aimed to compare postoperative blood loss and transfusion requirement between epidural PCA and intravenous PCA via propensity score-matching analysis in a large cohort of patients undergoing unilateral TKA.

## Results

### Patient characteristics and preoperative laboratory values

**Figure 1:**
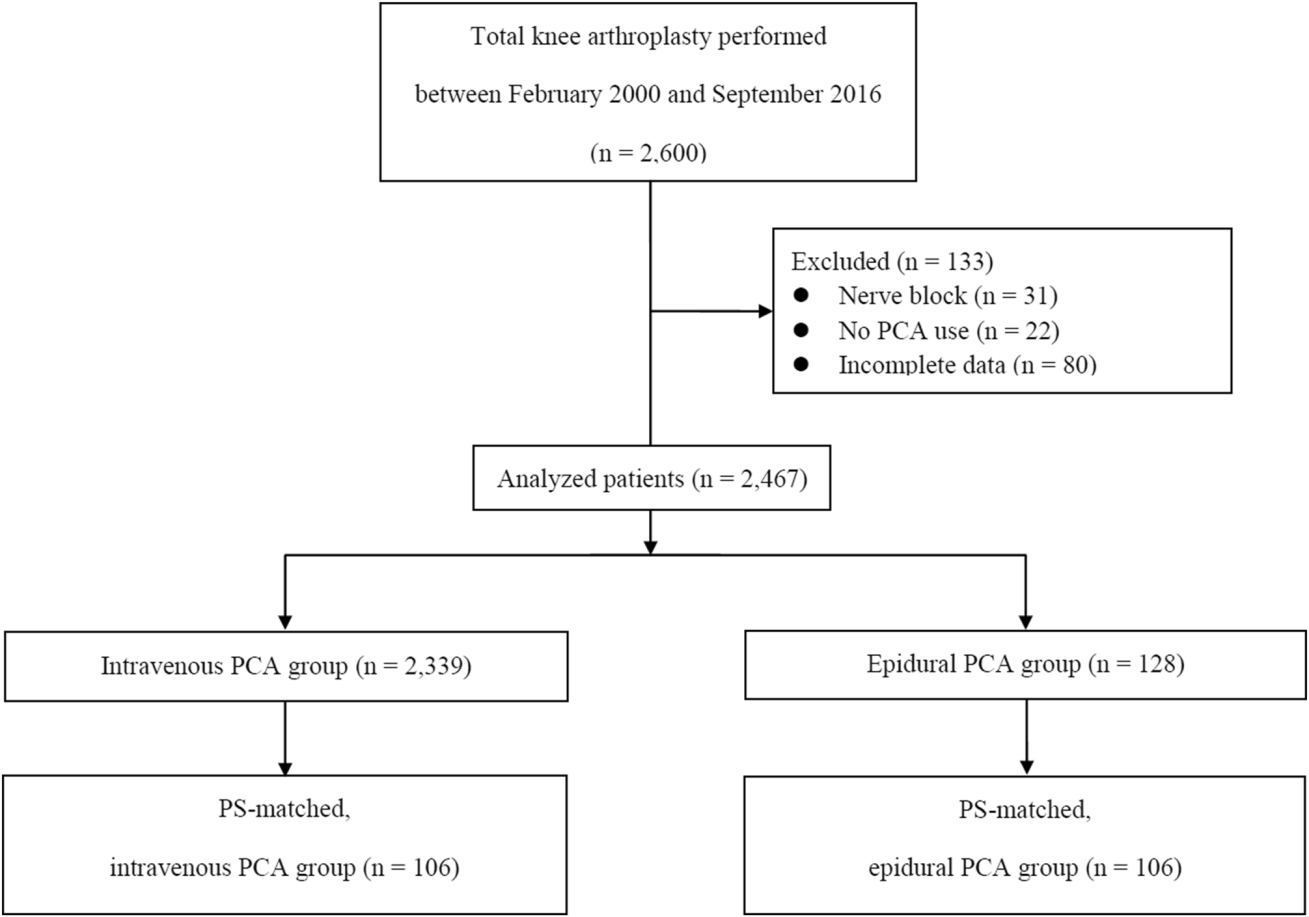
Study flow chart. PCA: patient-controlled analgesia, PS: propensity score.

A total of 2600 patients who underwent unilateral TKA between January 2000 and September 2016 were included in this study. We excluded those with peripheral nerve block (n = 31), those without continuous PCA use (n = 22), and incomplete data (n = 80). Accordingly, this study included 2467 patients who were divided into an intravenous PCA (n = 2339) or epidural PCA (n = 128) group (Fig. 1). The primary diagnosis and incidence of re-do TKA in both the PCA groups were not significantly different (Table 1). Table 1 demonstrates the preoperative laboratory values between the 2 groups.

**Table 1:** Unmatched preoperative characteristics and preoperative laboratory values between the intravenous PCA and epidural PCA groups.

Variables	Intravenous PCA (n = 2339)	Epidural PCA (n = 128)	*P v*alue
Gender, female (%)	2053 (87.8%)	112 (87.5%)	0.927
**ASA class**			
I/II	233(10.0%)/2019(86.3%)	10 (7.8%)/110 (85.9%)	< 0.001
III/IV	/87 (3.7%)/0	/7 (5.5%)/1 (0.8%)	
Age (years)	68.0 ± 7.6	68.4 ± 7.6	0.567
Weight (kg)	62.5 ± 9.6	61.7 ± 9.4	0.370
Height (cm)	153.6 ± 7.2	153.0 ± 7.0	0.359
Body mass index (kg/m^2^)	26.4 ± 3.4	26.3 ± 3.2	0.590
**Primary diagnosis**			
Osteoarthritis	2139 (91.4%)	122 (95.3%)	0.124
Rheumatoid arthritis	37 (1.6%)	0	0.152
Infectious arthritis	82 (3.5%)	2 (1.6%)	0.238
Traumatic knee injury	21 (0.9%)	2 (1.6%)	0.446
Ankylosing knee	4 (0.2%)	0	0.640
Others	65 (2.8%)	2 (1.6%)	0.410
Re-operation	207 (8.8%)	11 (8.6%)	0.921
**Preoperative laboratory values**			
Hemoglobin level (g/dL)	12.6 ± 1.3	12.7 ± 1.4	0.402
Platelet count (× 103/μL)	248.3 ± 64.8	251.7 ± 60.7	0.566
Prothrombin time (INR)	1.0 ± 2.1	1.0 ± 0.1	0.814
AST level (U/L)	23.6 ± 9.6	24.6 ± 11.4	0.213
ALT level (U/L)	19.9 ± 11.5	20.3 ± 14.0	0.701
ALP level (U/L)	75.9 ± 26.8	74.5 ± 25.1	0.560
Total bilirubin level (mg/dL)	0.7 ± 0.3	0.7 ± 0.2	0.160
Protein level (g/dL)	6.8 ± 0.5	6.9 ± 0.5	0.047
Albumin level (g/dL)	3.9 ± 0.3	3.8 ± 0.3	0.051
Creatinine level (mg/dL)	0.8 ± 0.5	0.9 ± 0.5	0.166
BUN level (mg/dL)	17.2 ± 5.8	17.5 ± 6.7	0.603
Uric acid level (mg/dL)	4.8 ± 1.3	5.0 ± 1.5	0.077
Glucose level (mg/dL)	128.7 ± 46.5	124.4 ± 38.2	0.299
Sodium level (mEq/L)	141.0 ± 2.5	141.5 ± 2.0	0.061
Potassium level (mEq/L)	4.1 ± 0.4	4.1 ± 0.4	0.781

Data are presented as mean ± standard deviation or number (%), as appropriate.

*PCA* patient-controlled analgesia, *ASA* American Society of Anesthesiologists, *INR* international normalized ratio, *AST* aspartate transaminase, *ALT* alanine transaminase, *ALP* alkaline phosphatase, *BUN* blood urea nitrogen. Others include avascular necrosis, desmoplastic fibroma, fibrous dysplasia, pigmented villonodular synovitis, spontaneous osteonecrosis, valgus knee, spastic diplegia of cerebral palsy, and fused knee.

### Intraoperative and postoperative variables

The patients in the intravenous PCA group were more likely to receive inhalation anesthesia (*P* < 0.001). In contrast, epidural PCA use was more frequent in patients who received regional anesthesia (*P* < 0.001) (Table 2). In total patients, 1992 (77.9%) patients underwent blood transfusion during intra- or postoperative period. Although the estimated blood loss during operation in the intravenous PCA group was greater than that in the epidural PCA group, the amount of red cell transfusion and fresh frozen plasma were not significantly different between the groups (Table 2). Other fluid administration data and urine output are presented in Table 2. The postoperative variables are provided in Table 3. Numerical rating scale (NRS) in maximal pain intensity at the postoperative anesthetic care unit and the ward on postoperative day 0 was significantly lower in epidural PCA group compared with intravenous PCA group (*p* < 0.001). The operation site drainage, total blood loss, and significant blood loss were greater in the epidural PCA group than in the intravenous PCA group (*P* < 0.001) (Table 3). In addition, blood loss after transfer to ward was greater than both during operation and in recovery room.

**Table 2:** Unmatched intraoperative variables between the intravenous PCA and epidural PCA groups.

Variables	Intravenous PCA (n = 2339)	Epidural PCA (n = 128)	*P v*alue
**Type of anesthesia**			
Inhalation	2031 (86.8%)	23 (18.0%)	< 0.001
Total intravenous	48 (2.1%)	0	0.102
Regional	260 (11.1%)	105 (82.0%)	< 0.001
**Intraoperative variable**			
Crystalloid use (mL)	974.9 ± 590.8	1165.7 ± 590.1	< 0.001
Colloid use (mL)	402.5 ± 291.0	219.9 ± 259.9	< 0.001
Packed red blood cell use (U)	0.3 ± 0.7	0.2 ± 0.5	0.621
Fresh frozen plasma use (U)	0.01 ± 0.20	0	0.602
Estimated blood loss (mL)	77.6 ± 198.7	29.7 ± 113.2	0.007
Operation site drainage (mL)	3.3 ± 60.8	0	0.538
Urine output (mL)	195.3 ± 226.8	316.1 ± 324.0	< 0.001

Data are presented as mean ± standard deviation or number (%), as appropriate.

*PCA* patient-controlled analgesia.

**Table 3:** Unmatched postoperative variables between the intravenous PCA and epidural PCA groups.

Variables	Intravenous PCA (n = 2339)	Epidural PCA (n = 128)	*P v*alue
Postoperative hypotension	268 (11.5%)	8 (6.3%)	0.069
Postoperative ICU admission	17 (0.7%)	3 (2.3%)	0.047
Re-admission	44 (1.9%)	1 (0.8%)	0.365
Hospital stay (day)	17.1 ± 9.2	15.4 ± 7.9	0.043
**Variables in recovery room**			
Crystalloid use (mL)	274.9 ± 271.1	245.2 ± 327.3	0.233
Colloid use (mL)	114.1 ± 251.6	80.5 ± 176.4	0.137
Packed red blood cell use (U)	0.2 ± 0.5	0.2 ± 0.4	0.966
Fresh frozen plasma use (U)	0.003 ± 0.078	0	0.687
Operation site drainage (mL)	45.4 ± 127.9	42.7 ± 141.2	0.817
Urine output (mL)	171.1 ± 174.4	167.2 ± 148.0	0.803
**Variables in postoperative ward**			
Packed red blood cell use (U)	1.8 ± 1.5	2.0 ± 1.3	0.120
Packed red blood cell use > 3 units	526 (22.5%)	38 (29.7%)	0.059
Fresh frozen plasma use (U)	0.04 ± 0.82	0.02 ± 0.18	0.770
Operation site drainage (mL)	704.3 ± 397.8	889.2 ± 385.9	< 0.001
Total blood loss (mL)	751.6 ± 451.9	931.9 ± 450.6	< 0.001
Significant blood loss	317 (13.6%)	30 (23.4%)	0.003
Maximal pain intensity (NRS)	5.05 ± 3.30	1.87 ± 3.30	< 0.001

Data are presented as mean ± standard deviation or number (%), as appropriate.

*PCA* patient-controlled analgesia, *ICU* intensive care unit. Postoperative hypotension = systolic blood pressure < 90 mmHg, diastolic blood pressure < 60 mmHg within postoperative day 3, NRS =  numerical rating scale.

### Results of propensity matching

All the variables of propensity score-matched patients (n = 212) are listed in Tables 4, 5 and 6. The American Society of Anesthesiologists (ASA) class, preoperative protein values, inhalation anesthesia use, regional anesthesia use, and intraoperative variables (crystalloid and colloid amounts, estimated blood loss, and urine output) significantly differed between the intravenous PCA (n = 2339) and epidural PCA (n = 128) groups before matching (Tables 2, 3). After propensity score-matching, the patient characteristics and preoperative and intraoperative values did not significantly differ between the groups (Tables 4, 5). Moreover, the incidence of postoperative admission to the intensive care unit and hospital stay did not significantly differ between the groups (Table 6). Similar to unmatched results, NRS in maximal pain intensity at the postoperative anesthetic care unit and the ward on postoperative day 0 was significantly lower in epidural PCA group compared with intravenous PCA group (*p* < 0.001), and blood loss after transfer to ward was greater than both during operation and in recovery room. The total amount of operation site bleeding during the postoperative period, and total blood loss in the epidural PCA group was greater than in the intravenous PCA group (900.9 ± 369.1 vs. 737.8 ± 410.1, *P* = 0.007; 996.7 ± 474.4 vs. 818.4 ± 447.8, *P* = 0.007, respectively). Packed red blood cell transfusion of > 3 units were also significantly more in the epidural PCA group compared with the intravenous PCA group [32 (30.2%) vs.17 (16.0%), *P* = 0.014]. The percentage of significant blood loss in the epidural PCA group was about twice over the intravenous PCA group [25(23.6%) vs. 13(12.3%), *P* = 0.052].

**Table 4:** Preoperative characteristics and preoperative laboratory values between the intravenous PCA and epidural PCA groups: PS-matched data.

Variables	Intravenous PCA (n = 106)	Epidural PCA (n = 106)	*P v*alue
Gender, female (%)	89 (84.0%)	92 (86.8%)	0.710
**ASA class**			
I/II	8 (7.5%)/93 (87.7%)	9 (8.5%)/91 (85.8%)	0.918
III/IV	/5 (4.7%)/0	/6 (5.7%)/0	
Age (years)	69.3 ± 7.4	68.7 ± 7.4	0.627
Weight (kg)	61.9 ± 9.3	61.6 ± 8.8	0.820
Height (cm)	154.3 ± 7.2	153.1 ± 7.2	0.235
Body mass index (kg/m^2^)	26.0 ± 3.2	26.2 ± 3.0	0.547
**Primary diagnosis**			
Osteoarthritis	103 (97.2%)	101 (95.3%)	0.727
Rheumatoid arthritis	0	0	–
Infectious arthritis	0	2 (1.9%)	0.500
Traumatic knee injury	1 (0.9%)	1 (0.9%)	1.000
Ankylosing knee	0	0	–
Others	4 (3.8%)	2 (1.9%)	0.687
Re-operation	9 (8.5%)	10 (9.4%)	1.000
**Preoperative laboratory values**			
Hemoglobin level (g/dL)	12.7 ± 1.4	12.7 ± 1.4	0.906
Platelet count (× 103/μL)	241.0 ± 62.5	252.5 ± 61.9	0.168
Prothrombin time (INR)	1.0 ± 0.1	1.0 ± 0.1	0.637
AST level (U/L)	23.2 ± 7.9	24.1 ± 11.9	0.520
ALT level (U/L)	18.6 ± 10.0	19.9 ± 14.7	0.486
ALP level (U/L)	76.6 ± 23.3	74.4 ± 25.9	0.552
Total bilirubin level (mg/dL)	0.6 ± 0.4	0.7 ± 0.2	0.182
Protein level (g/dL)	6.8 ± 0.4	6.9 ± 0.5	0.314
Albumin level (g/dL)	3.8 ± 0.3	3.8 ± 0.3	0.688
Creatinine level (mg/dL)	0.9 ± 0.8	0.8 ± 0.3	0.602
BUN level (mg/dL)	18.4 ± 6.7	17.6 ± 6.7	0.335
Uric acid level (mg/dL)	4.8 ± 1.3	1.9 ± 1.3	0.677
Glucose level (mg/dL)	125.1 ± 39.2	125.7 ± 38.6	0.912
Sodium level (mEq/L)	141.1 ± 2.7	141.4 ± 2.0	0.258
Potassium level (mEq/L)	4.2 ± 0.4	4.1 ± 0.4	0.421

Data are presented as mean ± standard deviation or number (%), as appropriate.

*PCA* patient-controlled analgesia, *PS* propensity score, *ASA* American Society of Anesthesiologists, *INR* international normalized ratio, *AST* aspartate transaminase, *ALT* alanine transaminase, *ALP* lkaline phosphatase, *BUN* blood urea nitrogen. Others include avascular necrosis, desmoplastic fibroma, fibrous dysplasia, pigmented villonodular synovitis, spontaneous osteonecrosis, valgus knee, spastic diplegia of cerebral palsy, and fused knee.

**Table 5:** Intraoperative variables between the intravenous PCA and epidural PCA groups: PS-matched data.

Variables	Intravenous PCA (n = 106)	Epidural PCA (n = 106)	*P v*alue
**Type of anesthesia**			
Inhalation	25 (23.6%)	23 (21.7%)	0.687
Total intravenous	0	0	–
Regional	81 (76.4%)	83 (78.3%)	0.687
**Intraoperative variable**			
Crystalloid use (mL)	1044.8 ± 548.9	1096.7 ± 543.2	0.386
Colloid use (mL)	254.2 ± 259.2	241.5 ± 268.9	0.677
Packed red blood cell use (U)	0.2 ± 0.6	0.2 ± 0.5	0.879
Fresh frozen plasma use (U)	0	0	–
Estimated blood loss (mL)	41.0 ± 121.7	32.1 ± 118.4	0.575
Operation site drainage (mL)	0	0	–
Urine output (mL)	272.9 ± 328.0	300.8 ± 313.9	0.521

Data are presented as mean ± standard deviation or number (%), as appropriate.

*PS* propensity score, *PCA* patient-controlled analgesia.

**Table 6:** Postoperative variables between the intravenous PCA and epidural PCA groups: PS-matched data.

Variables	Intravenous PCA (n = 106)	Epidural PCA (n = 106)	*P v*alue
Postoperative hypotension	8 (8.4%)	7 (7.4%)	1.000
Postoperative ICU admission	1 (1.1%)	3 (3.2%)	0.625
Re-admission	3 (3.2%)	1 (1.1%)	0.625
Hospital stay (day)	16.9 ± 7.1	15.1 ± 8.0	0.107
**Variables in recovery room**			
Crystalloid use (mL)	281.5 ± 375.5	264.2 ± 348.3	0.711
Colloid use (mL)	64.5 ± 116.5	88.7 ± 194.6	0.301
Packed red blood cell use (U)	0.2 ± 0.4	0.2 ± 0.4	0.356
Fresh frozen plasma use (U)	0	0	0.690
Operation site drainage (mL)	45.9 ± 125.1	42.1 ± 150.7	0.855
Urine output (mL)	167.9 ± 236.4	169.2 ± 148.2	0.965
**Variables in postoperative ward**			
Packed red blood cell use (U)	1.7 ± 1.5	1.9 ± 1.3	0.339
Packed red blood cell use > 3 units	17 (16.0%)	32 (30.2%)	0.014
Fresh frozen plasma use (U)	0	0.02 ± 0.21	0.320
Operation site drainage (mL)	737.8 ± 410.1	900.9 ± 369.1	0.007
Total blood loss (mL)	818.4 ± 447.8	996.7 ± 474.4	0.007
Significant blood loss	13 (12.3%)	25 (23.6%)	0.052
Maximal pain intensity (NRS)	5.42 ± 3.63	1.95 ± 3.31	< 0.001

Data are presented as mean ± standard deviation or number (%), as appropriate.

*PCA* patient-controlled analgesia, *PS* propensity score, *ICU* intensive care unit. Postoperative hypotension = systolic blood pressure < 90 mmHg, diastolic blood pressure < 60 mmHg within postoperative day 3, NRS =  numerical rating scale.

Using a univariate conditional logistic regression model, we found that the incidence of packed red blood cell transfusion of > 3 units was associated with epidural PCA (odds ratio, 2.5; 95% confidence interval, 1.201–5.205; *P* = 0.014) (Table 7).

**Table 7:** Conditional logistic regression analysis of epidural PCA predicting packed red blood cell transfusion more than 3 units.

Variables	Odds ratio	95% confidence interval	*P* value
Epidural PCA	2.500	1.201–5.205	0.014

*PCA* patient-controlled analgesia.

## Discussion

Our current analysis indicates that the amount of intraoperative bleeding was relatively small, compared to the amount of bleeding after unilateral TKA, as a result of tourniquet use during the operation. Therefore, the prevention and reduction of postoperative bleeding are more important than that of intraoperative bleeding. The allogenic blood transfusion can lead to a higher incidence of the respiratory tract and wound infection, greater in-hospital mortality, and longer hospital stay in total hip or knee surgery, therefore, the prevention and reduction of blood loss in patients undergoing knee surgery are crucial^7^. After the propensity score-matching of preoperative and intraoperative variables, we found that the amount of postoperative bleeding was greater in the epidural PCA group than in the intravenous PCA group. In addition, significant blood loss was more in the epidural PCA group compared with the intravenous PCA group despite being a marginally statistical significance (*P* = 0.052). To our knowledge, our present study is the first to investigate whether epidural PCA affects postoperative bleeding by controlling preoperative and intraoperative factors. Pöpping and colleagues demonstrated that epidural PCA did not show a significant relationship with the need for intraoperative and postoperative blood transfusion^14^. In our present study, the incidence of postoperative blood transfusion in the epidural PCA group was not significantly different, as compared to that in the intravenous PCA group. In contrast, some previous studies reported on the relationship between regional anesthesia and decreased blood loss^9–12^. These blood-sparing effects of regional anesthesia are believed to result from the diminished sympathetic tone of vessels in the surgical field and the indirect effect of concomitantly reduced arterial and venous pressure^12^. However, the effects of regional anesthesia on postoperative bleeding are inconsistent as the lumbar epidural block, which usually produces a sympathetic blockade below T10, results in minimal vasodilatory effects^15^.

There are considerable evidences that suggest an aggressive hypotensive strategy during surgery can reduce the blood loss associated with orthopedic procedures. However, the induction of hypotension during the operation is not currently recommended^16^. Bruce and colleagues reported that the appropriate evaluation of bleeding risk preoperatively, tourniquet use during surgery, maintenance of normothermia perioperatively, and use of antifibrinolytic agents were important measures for the prophylaxis of perioperative bleeding^16^. However, most of the studies to date that have evaluated the risk factors of postoperative bleeding in patients undergoing TKA have certain limitations, including unbalanced demographic data and intraoperative variables between groups, or a relatively small sample size in each group^13^. In particular, the significant predictors of transfusion, such as preoperative hemoglobin level, age, female gender, body mass index, creatinine level, intraoperative blood loss, and intraoperative fluid use, should be controlled before comparison. Some reports have demonstrated that there is a significant relationship between epidural PCA and bleeding tendency after surgery. Nielsen and colleagues reported that the reduction in stress response with epidural PCA might indirectly affect platelet dysfunction^17^. Similarly, Modig and colleagues reported that elevated fibrinolytic activity developed after epidural PCA administration, as compared to parenteral analgesia administration, for postoperative pain^18^.

Although postoperative infection after TKA is an infrequent complication, it is strongly related to patient morbidity and increased hospital costs. Several studies have described postoperative infection as a risk factor for TKA failure^19, 20^. Fehring and colleagues demonstrated that infection was one of the most frequent reasons for early failure, which requires revision of TKA^19^. The allogenic transfusion of blood products is known as a significant risk factor for postoperative infection. Chang and colleagues demonstrated that there is a significant dose-dependent relationship between transfusion and the infection rate^21^. Houbiers and colleagues demonstrated that the corrected relative risk for postoperative bacterial infection was 3.6 for a transfusion of more than 3 units^22^. In the present study, we found that patients who received epidural PCA were 2.5 times more likely to receive red cell transfusion of more than 3 units, as compared to patients who received intravenous PCA. We considered that it might be associated with significant blood loss. Hence, epidural PCA for postoperative pain management following TKA may increase postoperative bleeding and transfusion, which can consequently increase the occurrence of postoperative complications. Recently, peripheral nerve block technique such as continuous femoral-sciatic nerve blocks is an alternative option to epidural analgesia after TKA^23^. Moreover, it shows fewer complications over the epidural PCA. Therefore, given these considerations, epidural PCA should be carefully used for postoperative pain management in TKA.

Although we compared both groups with propensity score-matching analysis using variables obtained from retrospective studies, a randomized controlled study is warranted to identify the effect of epidural PCA on postoperative bleeding, in comparison with that of parenteral regimens. Appropriate pain management was initiated by a specialized acute pain management team at our institution, although we did not evaluate the between-serial measurements of the pain scores and the duration of PCA use during the postoperative period in the present study.

In summary, our current propensity matching analysis has indicated that epidural PCA is strongly related to postoperative bleeding and the incidence of transfusion of more than 3 units after unilateral TKA, as compared to that with intravenous PCA. Therefore, the use of epidural PCA may be carefully considered for postoperative pain management in TKA.

## Materials and methods

### Patient characteristics

Patients who underwent primary or revisional unilateral TKA between January 2000 and September 2016 in our institution were included. Patients who underwent bilateral TKA, patients with incomplete laboratory data or nerve block, and those without continuous PCA use were excluded. Two operators with > 10 years of experience in performing TKA performed all surgery. None of the study patients received an autologous blood transfusion or tranexamic acid during operation. All patients used tourniquets during surgery. The hemoglobin level was maintained > 8.0 g/dL. When the level of hemoglobin decreased to < 8 g/dL, packed red blood cell transfusion was started according to the anesthetic protocol of our hospital. Passive exercise and ambulation after TKA were performed according to the rehabilitation program of the orthopedic department.

### Clinical data collection

Demographic data, preoperative laboratory values, primary diagnosis, re-do TKA, anesthetic technique, type of PCA, intraoperative variables, recovery room variables, and postoperative variables were obtained from the electronic medical records system. The Demographic data included sex, age, weight, height, body mass index, the ASA physical status classification, and preoperative laboratory values. The anesthetic techniques were classified as inhalation, total intravenous, or regional anesthesia. The intraoperative variables included the total amount of each type of fluid (crystalloid and colloid), urine output, estimated blood loss, packed red blood cell use, fresh frozen plasma use, and operation site drainage amount. The recovery room variables included the total amount of each type of fluid (crystalloid and colloid), urine output, packed red blood cell use, fresh frozen plasma use, and operation site drainage amount. The postoperative variables included packed red blood cell use, fresh frozen plasma use, operation site drainage amount, total blood loss, and significant blood loss. The operation site drainage amount is the amount of pure blood in hemovac. Total blood loss is a sum of operation site drainages during the intraoperative and postoperative period, and estimated blood loss in the operation room. Significant blood loss is defined as a loss of above 30% of circulating the total blood volume^24^. We calculated estimated total blood volume (ETBV) according to Allen’s calculation: ETBV is 70 mL/kg for males or 65 mL/kg for females^25^. A total blood loss greater than 30% of the ETBV is considered significant blood loss. The pain intensity was assessed using the 11-point numerical rating scale (NRS; 0 = no pain, 10 = unbearable pain) by nurses. The highest NRS score in pain intensity was collected at the postoperative anesthetic care unit and the ward on postoperative day 0 were collected. The number of episodes of postoperative hypotension within postoperative day 3, the incidence of admission to the intensive care unit, total hospital stays, and incidence of re-admission for surgical complications were also recorded.

### Study outcomes

The primary outcome included the comparison of the postoperative blood loss between epidural PCA and intravenous PCA by using propensity score-matching analysis. The secondary outcome was to determine the relationship between the incidence of packed red blood cell transfusion of > 3 units and epidural PCA.

### Statistical analysis

Data are expressed as mean ± standard deviation, or number (percent), as appropriate. The data variables included in this study were compared between the epidural PCA and intravenous PCA groups using the chi-squared test or Fisher’s exact test for categorical variables and Student’s t-test or the Mann–Whitney U test for continuous variables. We performed multiple logistic regression analysis to determine the propensity score using the following variables: sex, age, body mass index, ASA physical status class, preoperative laboratory values (platelet count, prothrombin time, and aspartate transaminase, alanine transaminase, protein, albumin, serum creatinine, blood urea nitrogen, hemoglobin, and glucose levels), anesthesia technique, re-do TKA, primary diagnosis (osteoarthritis, rheumatoid arthritis, infectious arthritis, traumatic knee injury, and ankylosing knee), and intraoperative variables (crystalloid amount, colloid amount, estimated blood loss, and operation site drainage).

After performing 1:1 propensity score-matching, continuous variables were compared using the paired samples t-test or Wilcoxon signed-rank test, as appropriate, whereas categorical variables were compared using McNemar’s test or the marginal homogeneity test, as appropriate. Model calibration was assessed using Hosmer–Lemeshow statistics (χ2 = 8.996; df = 23; *P* = 0.996). We conducted univariate conditional logistic regression analysis for the matched population to identify the risk of blood transfusion. In all analyses, a *P* value of < 0.05 was considered statistically significant. Statistical analysis was conducted using R (version 3.1.2; R Foundation for Statistical Computing, Vienna, Austria). Conditional logistic regression analyses were performed with STATA Release 14 (StataCorp 2015; Stata Statistical Software, College Station, TX, USA).

### Ethics

This study was performed according to the Declaration of Helsinki. The current study protocol was approved by the institutional review board of Asan Medical Center, Seoul, Korea (approval number: 2016–1233). Due to the retrospective nature of the study, informed consent was waived.

## Data Availability

The datasets generated during and/or analysed during the current study are available from the corresponding author on reasonable request.

## References

[1] Perkins FM, Kehlet H (2000). Chronic pain as an outcome of surgery: a review of predictive factors. Anesthesiology.

[2] Liu S, Carpenter RL, Neal JM (1995). Epidural anesthesia and analgesia: their role in postoperative outcome. Anesthesiology.

[3] Kim SH, Yoon KB, Yoon DM, Kim CM, Shin YS (2013). Patient-controlled epidural analgesia with ropivacaine and fentanyl: experience with 2276 surgical patients. Korean J. Pain.

[4] Block BM (2003). Efficacy of postoperative epidural analgesia: a meta-analysis. JAMA.

[5] Singelyn FJ, Deyaert M, Joris D, Pendeville E, Gouverneur JM (1998). Effects of intravenous patient-controlled analgesia with morphine, continuous epidural analgesia, and continuous three-in-one block on postoperative pain and knee rehabilitation after unilateral total knee arthroplasty. Anesth. Analg..

[6] Shoji H, Solomonow M, Yoshino S, D'Ambrosia R, Dabezies E (1990). Factors affecting postoperative flexion in total knee arthroplasty. Orthopedics.

[7] Friedman R, Homering M, Holberg G, Berkowitz SD (2014). Allogeneic blood transfusions and postoperative infections after total hip or knee arthroplasty. J. Bone Joint Surg. Am..

[8] Klika AK (2014). Primary total knee arthroplasty allogenic transfusion trends, length of stay, and complications: nationwide inpatient sample 2000–2009. J Arthroplasty.

[9] Rosberg B, Fredin H, Gustafson C (1982). Anesthetic techniques and surgical blood loss in total hip arthroplasty. Acta Anaesthesiol. Scand..

[10] Twyman R, Kirwan T, Fennelly M (1990). Blood loss reduced during hip arthroplasty by lumbar plexus block. J. Bone Joint Surg. Br..

[11] Juelsgaard P, Larsen UT, Sorensen JV, Madsen F, Soballe K (2001). Hypotensive epidural anesthesia in total knee replacement without tourniquet: reduced blood loss and transfusion. Reg. Anesth. Pain. Med..

[12] Stevens RD, Van Gessel E, Flory N, Fournier R, Gamulin Z (2000). Lumbar plexus block reduces pain and blood loss associated with total hip arthroplasty. Anesthesiology.

[13] Frisch NB (2014). Predictors and complications of blood transfusion in total hip and knee arthroplasty. J. Arthroplasty.

[14] Popping DM (2014). Impact of epidural analgesia on mortality and morbidity after surgery: systematic review and meta-analysis of randomized controlled trials. Ann. Surg..

[15] Veering BT, Cousins MJ (2000). Cardiovascular and pulmonary effects of epidural anaesthesia. Anaesth. Intensive Care.

[16] Bruce W, Campbell D, Daly D, Isbister J (2013). Practical recommendations for patient blood management and the reduction of perioperative transfusion in joint replacement surgery. ANZ J. Surg..

[17] Nielsen TH (1989). Stress response and platelet function in minor surgery during epidural bupivacaine and general anaesthesia: effect of epidural morphine addition. Eur. J. Anaesthesiol..

[18] Modig J, Borg T, Bagge L, Saldeen T (1983). Role of extradural and of general anaesthesia in fibrinolysis and coagulation after total hip replacement. Br. J. Anaesth..

[19] Fehring TK, Odum S, Griffin WL, Mason JB, Nadaud M (2001). Early failures in total knee arthroplasty. Clin. Orthop. Relat. Res..

[20] Sharkey PF, Hozack WJ, Rothman RH, Shastri S, Jacoby SM (2002). Insall Award paper: Why are total knee arthroplasties failing today?. Clin. Orthop. Relat. Res..

[21] Chang H (2000). Allogeneic red blood cell transfusion is an independent risk factor for the development of postoperative bacterial infection. Vox Sang.

[22] Houbiers JG (1997). Transfusion of red cells is associated with increased incidence of bacterial infection after colorectal surgery: a prospective study. Transfusion.

[23] Kopp SL (2017). Anesthesia and analgesia practice pathway options for total knee arthroplasty: an evidence-based review by the american and european societies of regional anesthesia and pain medicine. Reg. Anesth. Pain Med..

[24] Manning JE, Kelen GD, Stapczynski JS (2003). Tintinalli's Emergency Medicine: A Comprehensive Study Guide.

[25] Hilberath JN (2015). Blood volumes in cardiac surgery with cardiopulmonary bypass. Perfusion.

